# DL-3-n-butylphthalide (NBP) alleviates poststroke cognitive impairment (PSCI) by suppressing neuroinflammation and oxidative stress

**DOI:** 10.3389/fphar.2022.987293

**Published:** 2023-01-11

**Authors:** Hui Zhang, Laifa Wang, Yongping Yang, Chuanhai Cai, Xueqin Wang, Ling Deng, Binsheng He, Wenhu Zhou, Yanhui Cui

**Affiliations:** ^1^ Neuroscience and Behavioral Research Center, Academician Workstation, Changsha Medical University, Changsha, China; ^2^ Hunan Key Laboratory of the Research and Development of Novel Pharmaceutical Preparations, Changsha Medical University, Changsha, China; ^3^ Xiangya School of Pharmaceutical Sciences, Central South University, Changsha, Hunan, China

**Keywords:** Dl-3-n-butylphthalide, neuroprotectants, acute ischemic stroke, inflammation, stress oxidation

## Abstract

Currently, the recovery of cognitive function has become an essential part of stroke rehabilitation. DL-3-n-butylphthalide (NBP) is a neuroprotective reagent and has been used in stroke treatment. Clinical studies have confirmed that NBP can achieve better cognitive outcomes in ischemic stroke patients than in healthy controls. In this study, we aimed to investigate the influences of NBP on cognitive function in an ischemic reperfusion (I/R) rat model. Our results showed that NBP profoundly decreased neurological scores, reduced cerebral infarct areas and enhanced cerebral blood flow (CBF). NBP potently alleviated poststroke cognitive impairment (PSCI) including depression-like behavior and learning, memory and social cognition impairments, in I/R rats. NBP distinctly suppressed the activation of microglia and astrocytes and improved neuron viability in the ischemic brain. NBP inhibited the expression of inflammatory cytokines, including interleukin-6 (IL-6), interleukin-1β (IL-1β) and tumor necrosis factor-α (TNF-α), by targeting the nuclear factor kappa B/inducible nitric oxide synthase (NF-κB/iNOS) pathway and decreased cerebral oxidative stress factors, including reactive oxygen species (ROS) and malondialdehyde (MDA), by targeting the kelch like ECH associated protein 1/nuclear factor-erythroid 2 p45-related factor 2 (Keap1/Nrf2) pathway in the ischemic brain. The current study revealed that NBP treatment improved neurological function and ameliorated cognitive impairment in I/R rats, possibly by synergistically suppressing inflammation and oxidative stress.

## Introduction

Currently, stroke is still the leading cause of disability and the second most common of mortality worldwide (Mendelson and Prabhakaran, 2021). Stroke is generally classified into hemorrhagic stroke and ischemic stroke and the latter accounts for 71% of all stroke cases (Sacks et al., 2018). Functional outcomes of stroke involve not only physical disability, but also cognitive impairment in approximately 1/3 of patients, severely affecting their capability to live independently (Kalaria et al., 2016). Poststroke cognitive impairment (PSCI) is a type of vascular cognitive impairment and likely develops into dementia (Teng et al., 2017). PSCI is quite common in acute ischemic stroke patients, with an incidence ranging from 7.4% to 41.3% (Huang et al., 2022). PSCI, including memory and learning disorders, emotional disorders, attention disorders, sensory and perceptual disorders, executive dysfunction, personality changes and behavioral abnormalities, has become one of the primary challenges faced in stroke rehabilitation (Liu et al., 2020). Lesions in cerebral areas, such as the hippocampus, white matter, and cortex, induced by ischemic reperfusion (I/R) might contribute to the pathogenesis of PSCI (Sun et al., 2014). Currently, drugs available for PSCI treatment mainly include calcium antagonists, excitatory amino acid receptor antagonists and cholinesterase inhibitors (Grupke et al., 2015). However, the effects of these reagents are still far from satisfactory, necessitating the development of novel preventive approaches to improve the cognitive function of AIS patients.

DL-3-n-butylphthalide (NBP) is a chiral compound synthesized from L-3-n-butylphthalide, which was originally extracted from the seeds of *Apium graveolens* Linn (Fan et al., 2022). NBP was licensed by the State Food and Drug Administration of China (SFDA) in 2002 for ischemic stroke treatment (Peng et al., 2013). Fundamental or clinical studies have demonstrated that NBP could alleviate PSCI (Sun et al., 2017; Yan et al., 2017). Multiple mechanisms are involved in the neuroprotective function of NBP, such as inhibiting neuroinflammation (Wang et al., 2019), resisting oxidative stress (Wang et al., 2021), promoting cerebral blood flow (CBF) (Li et al., 2019), and reducing neuronal damage (Li H Q et al., 2021). Oxidative stress and inflammation play essential roles in the pathogenesis of cognitive deficits induced by various risk factors (Tiwari et al., 2009; Tiwari and Chopra, 2012). Increasing evidence has suggested that neuroinflammation is an important factor contributing to PSCI, and the relevant mechanisms include neuronal cell injury, brain function impairment and inflammasome activation (Gold et al., 2011; Swardfager et al., 2013; Kim et al., 2020). Oxidative stress is another major mechanism participating in the pathogenesis of PSCI. Augmenting of oxidative stress levels damages neurons and contributes to aggravated PSCI (Bahader et al., 2021). Both neuroinflammation and oxidative stress provide targets for PSCI treatment (Zhang and Bi, 2020). In the present study, we hypothesized that NBP protects neurological function and alleviates PSCI, possibly by synergistically suppressing inflammation and oxidative stress. Therefore, the influences of NBP on neurological function and PSCI were investigated, and the underlying mechanisms were further probed.

## Materials and methods

### Ischemic reperfusion (I/R) model

Animal experiments were licensed by the Institutional Animal Care and Use Committee of Changsha Medical University. Animal suffering was minimized according to the guidelines. Sprague-Dawley (SD) rats with body weights ranging from 250 g to 280 g were purchased from Hunan SJA Laboratory (Changsha, China). The rats were housed in a room with a temperature of 20°C–24°C, humidity of 45%–65% and 12-h light/dark cycle.

**FIGURE 1:**
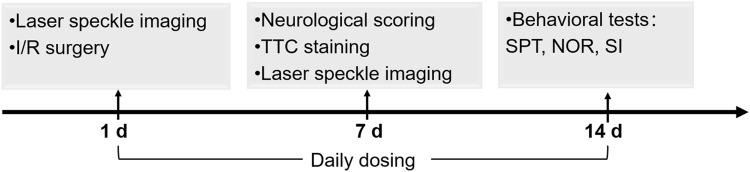
Schedule of the animal experiments including I/R model establishment, NBP dosing, cerebral infarction analysis, CBF analysis, neurological scoring and behavioral tests.

I/R models were established by middle cerebral artery occlusion (MCAO) according to Longa’s method (Longa et al., 1989) with minor modifications. The rats were anesthetized by pentobarbital sodium (30 mg/kg i.p.,). The right common carotid artery, external carotid artery (ECA) and internal carotid artery (ICA) were carefully separated and exposed. The common carotid artery was first ligated. A small incision was made at the ECA, and a Nylon suture with a .36-mm diameter (Cinontech, Beijing, China) was inserted and guided through the ICA to the middle cerebral artery (MCA). After 1 h of occlusion, the suture was withdrawn from the ICA for reperfusion. The sham group was subjected to a surgical procedure similar to that of the I/R group except for MCA occlusion. The rats in the vehicle + I/R group (I/R group) were administered saline (450 μL, i. p.), and the rats in the NBP + I/R group were administered NBP (9 mg/kg, i. p.) (CSPC Pharmaceutical Group Co., Ltd., Shijiazhuang, China). The flowchart of the animal experiment is generalized in Figure 1.

### Neurological deficit scoring

The neurological deficit score was evaluated on the 7th day after surgery by researchers blinded to the animal grouping. No deficit was scored 0; forelimb weakness was scored 1; circling to one side was scored 2; inability to bear weight on the affected side was scored 3; and no spontaneous motor activity was scored 4 (Shah et al., 2006). Rats scored 0 after I/R surgery were deemed failed models and were excluded from subsequent experiments.

### Laser speckle imaging

Laser speckle imaging (RWD Life Technologies, Shenzhen, China) was utilized to monitor CBF before MCAO, after MCAO, after reperfusion and on the 7th day after I/R surgery. Briefly, a scalp incision was made in the middle of the head. Bilateral skulls were ground and polished by a cranial perforator until the scull was sufficiently thin for CBF detection. The detector was positioned above the skull. Videos were obtained, and CBF in the region of interest (ROI) was recorded. Relative CBF for each rat was expressed as the ratio of CBF in the right hemisphere to the left hemisphere.

### Infarct area analysis

The cerebral infarct area was evaluated by 2,3,5-triphenyltetrazolium chloride (TTC) staining (Solarbio, Beijing, China). Briefly, the rats were anesthetized and sacrificed by cervical dislocation. Brains were rapidly removed and cut into slices of approximately 2-mm thickness on ice. The slices were immersed in TTC solution for 30 min at 37°C and fixed in 4% paraformaldehyde for 2 h. Photos of the slices were obtained. The infarct area was measured by ImageJ software (Media Cybernetics, Bethesda, MD, United States). The infarct volume was calculated through multiplying the area by the thickness of the slices. The percentage of the infarct volume was calculated.

### Sucrose preference test (SPT)

The SPT was performed to evaluate depression-like behavior (Lu et al., 2017). During the SPT procedure, the rats were kept separately. In the adaptation phase, each rat was supplied with two bottles of 1% (w/v) sucrose. After 24 h, one bottle of sucrose was replaced with water for another 24 h. Subsequently, the rat was deprived of water for 24 h. In the test phase, the rat was simultaneously supplied with one bottle of water and one bottle of 1% sucrose for 2 h. Sucrose preference (SP) was calculated according to the following formula: SP = sucrose intake/(sucrose intake + water intake) × 100%.

### Novel object recognition (NOR) test

The NOR test was performed to evaluate animal learning and memory capability (Lueptow, 2017). In the adaptive phase, two identical objects were placed in a square opaque box (20 cm × 20 cm). The rat was allowed to acclimate to the box for 5 min and then removed from the box to its home cage. In the test phase, one of the objects was replaced with a novel object with different materials, shapes and colors. The rat was returned to the box and allowed to freely explore for 5 min. The time spent sniffing or climbing each object by the rat was analyzed by Smart software, version 3.0 (Panlab, Spain). The Discrimination ratio was calculated according to the following formula (Time _Novel_—Time _Familiar_)/(Time _Novel_ + Time _Familiar_).

### Social interaction (SI) test

The SI test was performed to evaluate animal social cognitive capability (Liu et al., 2019). The device consisted three opaque square boxes (40 × 40 × 40 cm), with a passage connecting the boxes. One clear cage of the same size, sufficiently large to hold a rat, was positioned in the left box and the right box. In the first phase, the three boxes were separated with clear Plexiglas, and the subject rat was placed in the middle box to acclimate for 5 min. In the second phase, a stimulus rat (familiar rat) was placed to the left cage. The Plexiglas was removed to allow the subject rat to explore freely in the three boxes for 10 min. In the third phase, a second rat (unfamiliar rat) was placed in the right cage. The subject rat was allowed to freely explore for another 10 min. The time spent communicating with the familiar rat and the unfamiliar rat by the subject rat was analyzed by Smart software, version 3.0. The Discrimination ratio was calculated according to the following formula (Time _Unfamiliar_—Time _Familiar_)/(Time _Unfamiliar_ + Time _Familiar_) × 100%.

### Enzyme-linked immunosorbent assay (ELISA)

Inflammatory cytokines, including interleukin-6 (IL-6), interleukin-1β (IL-1β) and tumor necrosis factor-α (TNF-α), in the ischemic cerebral hemisphere were analyzed by ELISA kits according to the manufacturer’s instructions (Meiman, Jiangsu, China). A dose of 10 μL of supernatants of ischemic brain homogenate or standard sample was mixed with 40 μL of sample diluent and added to the wells. A dose of 100 μL of HRP-conjugated reagent was added to each well except for the blank wells. The plate was incubated at 37°C for 1 h. Chromogen Solution A (50 μL) and Chromogen Solution B (50 μL) were added to each well. The plate was incubated at 37°C for 15 min in the dark. The absorbance was measured at 450 nm.

### Reactive oxygen species (ROS) and malondialdehyde (MDA) analysis

The ischemic hemisphere of the brain was collected and homogenized. The homogenate was centrifuged at 4000 × g at 4°C for 20 min. The supernatant was collected. The protein concentration was determined by a BCA Protein Assay Kit (Kangwei Century Biotechnology, Jiangsu, China).

ROS were measured by an ROS assay kit (Jiancheng Bioengineering Institute, Nanjing, China) according to the manufacturer’s instructions. Briefly, 1 μL of sample, 5 μL of DCFH probe (5 μM) and PBS were mixed into a 96-well plate and incubated at 37°C for 40 min. The fluorescent value was measured under an excitation wavelength of 500 nm and an emission wavelength of 525 nm. The ROS level was expressed as a fold change in the fluorescent value compared with the sham group.

The MDA level was measured by an MDA assay kit (Jiancheng Bioengineering Institute, Nanjing, China) according to the manufacturer’s instructions. The test mixture contained 20 μL of sample, 20 μL of solution I, 1 mL of solution II and 330 μL of solution III. The blank mixture contained 20 μL of ethanol instead of sample and the standard mixture contained 20 μL standard substance (10 nM) instead of sample, with the other components the same. All of the mixtures were incubated in 95°C water bath for 40 min and centrifuged at 1000 × g for 10 min. The absorbance value at 532 nm was recorded. The MDA level was expressed as: OD _Sample_ × 10 nM/(OD _Standard_—OD _Blank_)/protein concentration (nM/mgprot).

### Western blot

The proteins extracted from the ischemic brain (10 μg per lane) were separated by 10% SDS‒PAGE gel and transferred to a .45 μm nitrocellulose membrane (Boster Biological Technology, CA, United States). The membranes were blocked with 5% nonfat milk at room temperature for 1 h and incubated with primary antibodies at 4°C overnight. After washing with TBST buffer 3 times, the membrane was incubated with HRP-conjugated secondary antibody at room temperature for 70 min. The bands were detected by an Odyssey Clx system (Li-COR Biosciences, United States). The protein expression level was quantified by ImageJ software and normalized to GAPDH. The primary antibodies included anti-Keap1 antibody (1:1000, ab119403, Abcam, United States), anti-Nrf2 antibody (1:500, ab92946, Abcam, United States), anti-NF-кB P65 antibody (1:500, ab194726, Abcam, United States), anti-iNOS antibody (1:500, ab283655, Abcam, United States), and anti-GAPDH antibody (1:2000, 10494-1-AP, ProteinTech, United States).

### Immunohistochemistry

The rats were anesthetized and perfused with saline solution and 4% paraformaldehyde. The brain was removed and sequentially immersed in 4% paraformaldehyde, 15% sucrose, 30% sucrose and 35% sucrose, each step for 12 h and imbedded into Tissue-Tek^®^ O.C.T (Sakura Finetek, United States). The brains were cut into slices with a thickness of 15 μm. The slices were incubated with primary antibodies at 4°C overnight. After washing with PBS, the slices were incubated with donkey anti-rabbit and donkey anti-mouse secondary antibodies at room temperature for 2 h. Subsequently, the slices were stained with 3,3′-diaminobenzidine (DAB) (ZSGB-BIO, Beijing, China) and washed with PBS. The primary antibodies included anti-NeuN antibody (1:200, ab177487, Abcam, United States), anti- GFAP polyclonal antibody (1:200, ab7260, Abcam, United States), and anti-Iba-1 antibody (1:200, 019-19741, Wako, Japan). The slides were dehydrated by ethanol, cleared by xylene and sealed. The same zone of brain sections was scanned using a Panoramic 250 FLASH II digital slide scanner (3DHISTECH, Budapest, Hungary). The numbers of positive cells per field were counted (×40 magnification). At least three sections for each rat were analyzed.

### Statistical analysis

The data are presented as the mean ± SEM and were analyzed by Graphpad Prism software, version 8 (San Diego, CA, United States). Group comparisons were performed by one-way ANOVA followed by Tukey’s *post hoc* test. *p* < .05 was considered significantly different.

## Results

### Effects of NBP on neurological scores, cerebral infarction and CBF in I/R rats

**FIGURE 2:**
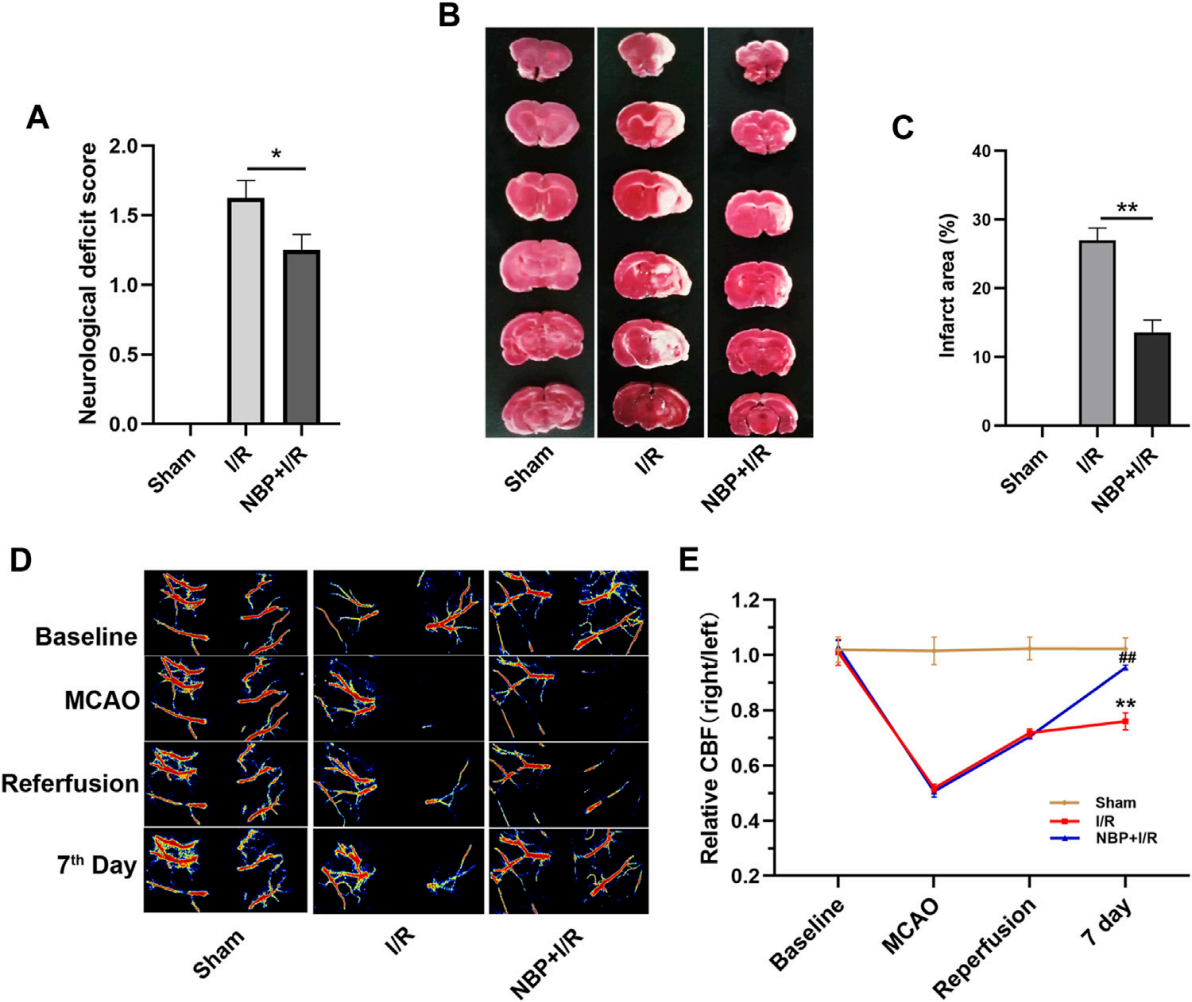
Effects of NBP on neurological scores, cerebral infarction and CBF in I/R rats. **(A)** Effects of NBP on neurological scores (n = 16). Rats showed obvious neurological deficits after I/R surgery, while NBP treatment significantly improved neurological function. **(B and C)** Effects of NBP on the infarct area in the right hemisphere of the brains in I/R rats (n = 3). I/R surgery resulted in obvious infarction in the right hemisphere of the brain, while NBP significantly reduced the infarct area in I/R rats. **(D and E)** Effects of NBP on CBF and the ratio of CBF in the right hemisphere to the left hemisphere (n = 3). Data are expressed as the mean ± SEM and were analyzed by one-way ANOVA (*post hoc* analysis: Tukey’s *post hoc* analysis test). * (*p* < .05) and ** (*p* < .01) represent significance compared to the I/R group. ## (*p* < .01) represents significance compared to the sham group. NBP dose: NBP + I/R, 9 mg/kg.

Neurological function was evaluated on the 7th day after I/R surgery. As shown in Figure 2A, the rats in the I/R group showed obvious neurological deficits compared with the sham group. NBP significantly improved neurological function (*p* < .05). TTC staining results showed that an obvious infarct area was observed in the I/R group, while NBP significantly reduced the infarct area in I/R rats (*p* < .01) (Figures 2B, C). CBF was monitored during I/R surgery and, on the 7th day after I/R surgery, by laser speckle imaging. CBF in the ischemic hemisphere of I/R rats was obviously reduced after MCAO and partially recovered after reperfusion, indicative of successful cerebral blood occlusion and reperfusion. On the 7th day, NBP significantly enhanced the CBF in the ischemic hemispheres of I/R rats (*p <* .01) (Figures 2D, E).

### Effects of NBP on cognitive behavior in I/R rats

**FIGURE 3:**
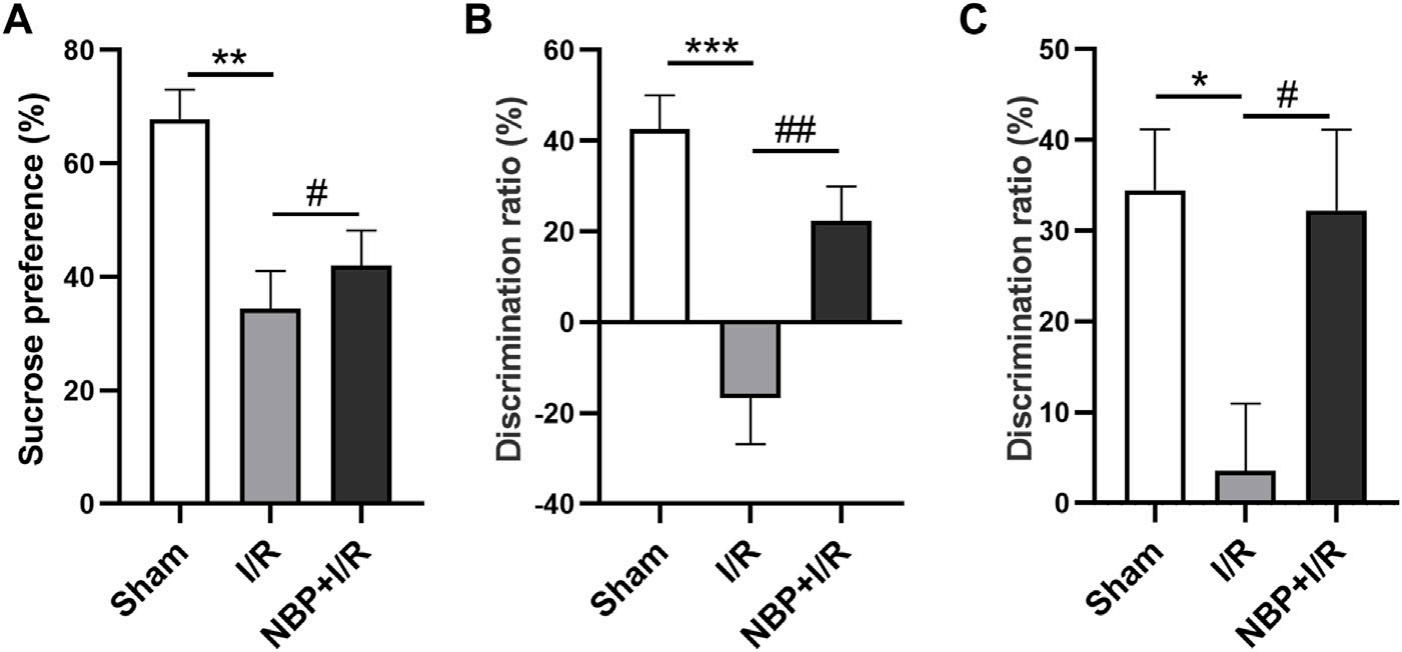
Effects of NBP on the cognitive behavior of I/R rats. **(A)** Effects of NBP on depression-like behavior in I/R rats as assessed by the SPT. Sucrose consumption was significantly decreased in I/R rats, while NBP significantly enhanced sucrose preference. **(B)** Effects of NBP on social cognition in I/R rats by SI testing. The discrimination ratio was significantly decreased in the I/R group and significantly increased after NBP treatment on the SI test. **(C)** Effects of NBP on study and memory in I/R rats by the NOR test. The discrimination ratio was significantly decreased in the I/R group and significantly increased after NBP treatment on the NOR test. Data are expressed as the mean ± SEM (n = 10) and were analyzed by one-way ANOVA (*post hoc* analysis: Tukey’s *post hoc* analysis test). * (*p* < .05), ** (*p* < .01) and *** (*p* < .001) represent significance compared to the sham group. # (*p* < .05) and ## (*p* < .01) represent significance compared to the I/R group (*p* < .01). NBP dose: NBP + I/R, 9 mg/kg.

The sucrose consumption by I/R rats was significantly decreased (*p* < .01). NBP significantly enhanced sucrose preference in I/R rats (*p* < .05) (Figure 3A), indicating that NBP could alleviate depression-like behavior in I/R rats. The SI test was performed to evaluate animal social cognition. Compared with the sham group, the discrimination ratio in the I/R group was significantly decreased (*p* < .001). NBP treatment significantly increased the discrimination ratio (*p* < .05), indicating that NBP could improve the social cognitive ability of I/R rats (Figure 3B). The NOR test was performed to investigate animal learning and memory capability. Compared with the sham group, the I/R group exhibited a significantly decreased discrimination ratio (*p* < .001). NBP treatment significantly increased the discrimination ratio compared with the I/R group (*p* < .05), suggesting that NBP could effectively improve the learning and memory abilities of I/R rats (Figure 3C).

### Effects of NBP on oxidative stress in I/R rats

**FIGURE 4:**
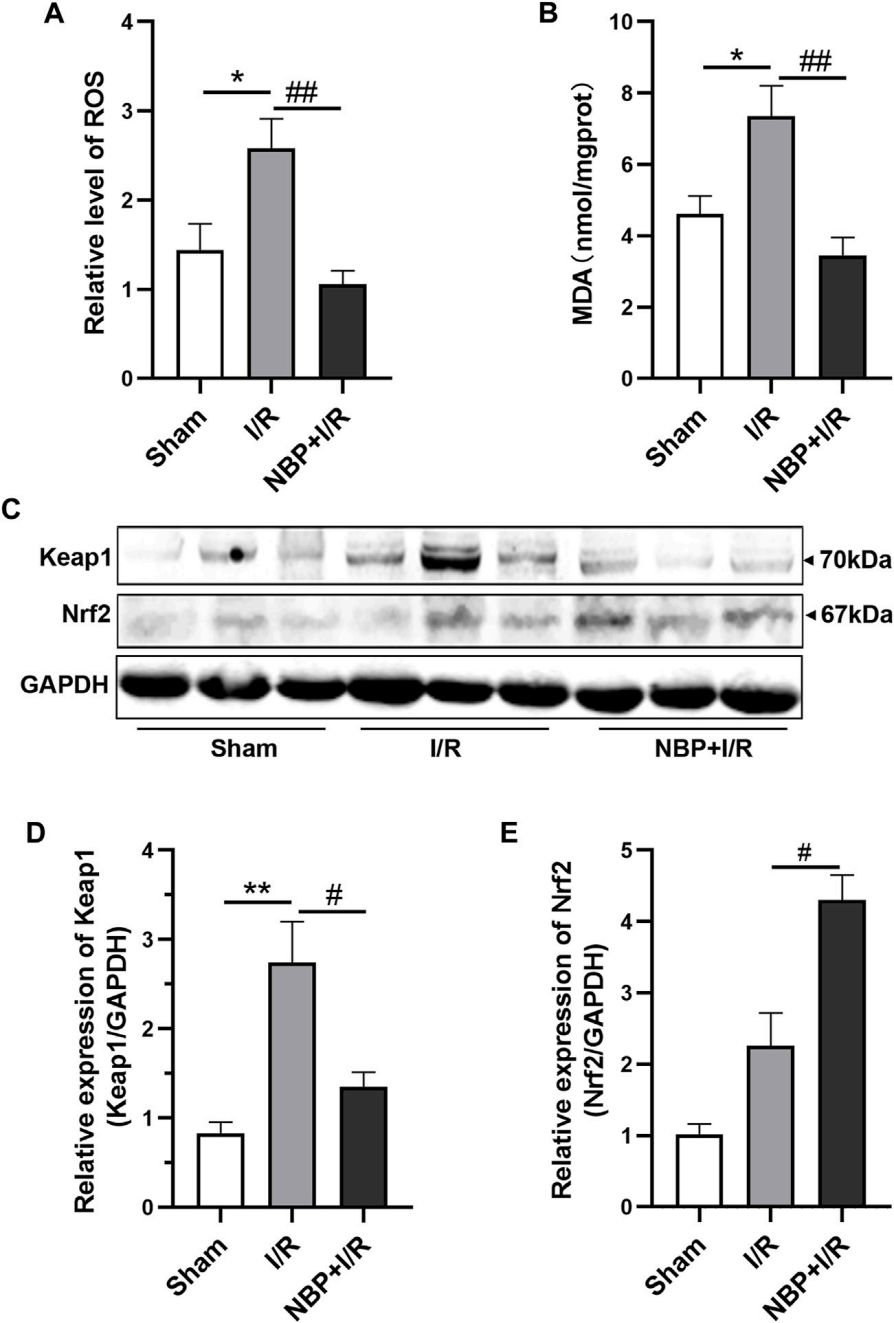
Effects of NBP on oxidative stress in the infarcted cerebral hemisphere in I/R rats. **(A and B)** Effects of NBP on ROS levels and MDA leves (n = 6). I/R surgery significantly elevated ROS levels and MDA levels, while NBP significantly decreased ROS levels and MDA levels in I/R rats. **(C–E)** Effects of NBP on Keap1/Nrf2 pathway (n = 3). NBP significantly downregulated Keap1 expression and upregulated Nrf2 expression in I/R rats. Data are expressed as the mean ± SEM and were analyzed by one-way ANOVA (*post hoc* analysis: Tukey’s *post hoc* analysis test). * (*p* < .05) and ** (*p* < .01) represent significance compared to the sham group. # (*p* < .05) and ## (*p* < .01) represent significance compared to the I/R group (*p* < .01). NBP dose: NBP + I/R, 9 mg/kg.

To evaluate the effects of NBP on oxidative stress, the levels of ROS and MDA in the right hemispheres of the rats were measured. The results suggested that transient ischemia significantly elevated ROS levels (*p* < .05) and MDA levels (*p* < .05). ROS (*p* < .01) and MDA (*p* < .01) were significantly decreased in the NBP + I/R group compared with the I/R group, indicating that NBP could suppress oxidative stress in I/R rats (Figures 4A, B). The oxidative stress-associated kelch like ECH associated protein 1/nuclear factor-erythroid 2 p45-related factor 2 (Keap1/Nrf2) pathway was further analyzed. The expression of both Keap1 and Nrf2 was low in the sham group. Keap1 protein expression was significantly elevated in the I/R group (*p* < .01). NBP significantly downregulated Keap1 levels (*p* < .05) and upregulated Nrf2 levels (*p* < .05), suggesting that NBP alleviated oxidative stress by targeting the Keap1/Nrf2 pathway (Figures 4C, D).

### Effects of NBP on neuroinflammation in I/R rats

**FIGURE 5:**
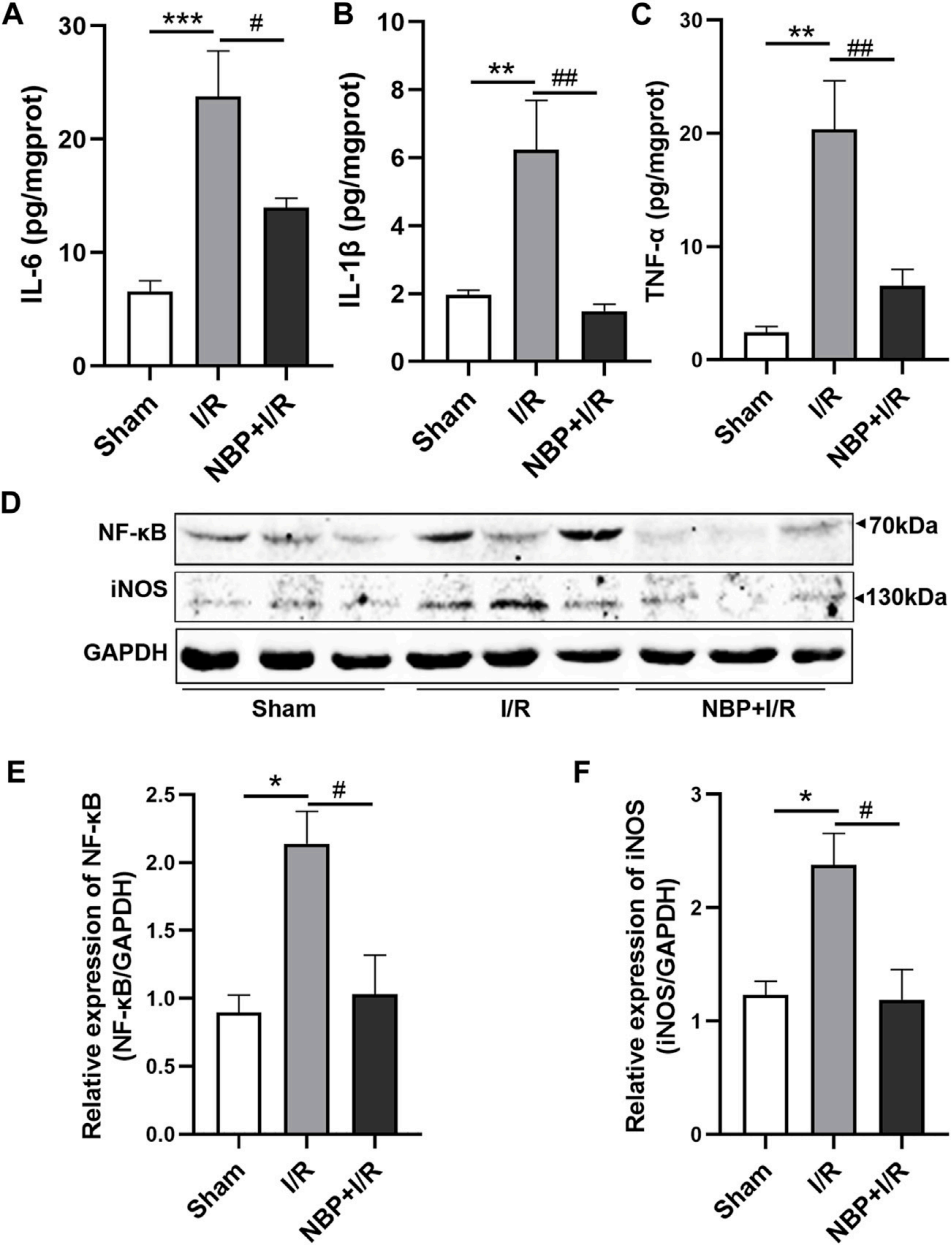
Effects of NBP on inflammation on the infarcted cerebral hemisphere in I/R rats. **(A–C)** Effects of NBP on inflammatory cytokines including IL-6, IL-1β and TNF-α. The concentrations of the three cytokines were significantly increased after I/R surgery and significantly decreased after NBP treatment. **(D–F)** Effects of NBP on the NF-κB/iNOS pathway. Data are expressed as the mean ± SEM and were analyzed by one-way ANOVA (*post hoc* analysis: Tukey’s *post hoc* analysis test). ^*^ (*p* < .05), ^**^ (*p* < .01) and ^***^ (*p <* .001) represent significance compared to the sham group. ^#^ (*p* < .05) and ^##^ (*p* < .01) represent significance compared to the I/R group (*p* < .01). NBP dose: NBP + I/R, 9 mg/kg.

Cytokines including IL-6, IL-1β and TNF-α, in the ischemic brain were assayed to investigate the effects of NBP on inflammatory levels in I/R rats. The concentrations of cerebral cytokines (IL-6, *p* < .01; IL-1β, *p* < .01; TNF-α, *p* < .01) were significantly increased after transient ischemia. NBP significantly decreased IL-6 (*p* < .01), IL-1β (*p* < .001) and TNF-α (*p* < .01) levels in the ischemic brains of the I/R rats (Figures 5A–C). The nuclear factor kappa B/inducible nitric oxide synthase (NF-κB/iNOS) was further analyzed. The expression of both NF-κB P65 (*p* < .05) and iNOS (*p* < .05) was significantly increased in the I/R group. NBP significantly downregulated NF-κB P65 (*p* < .05) and iNOS expression (*p* < .05). The above data suggested that NBP attenuated neuroinflammation by targeting the NF-κB/iNOS pathway (Figures 5D–F).

### Effects of NBP on neurons, microglia and astrocytes in I/R rats

**FIGURE 6:**
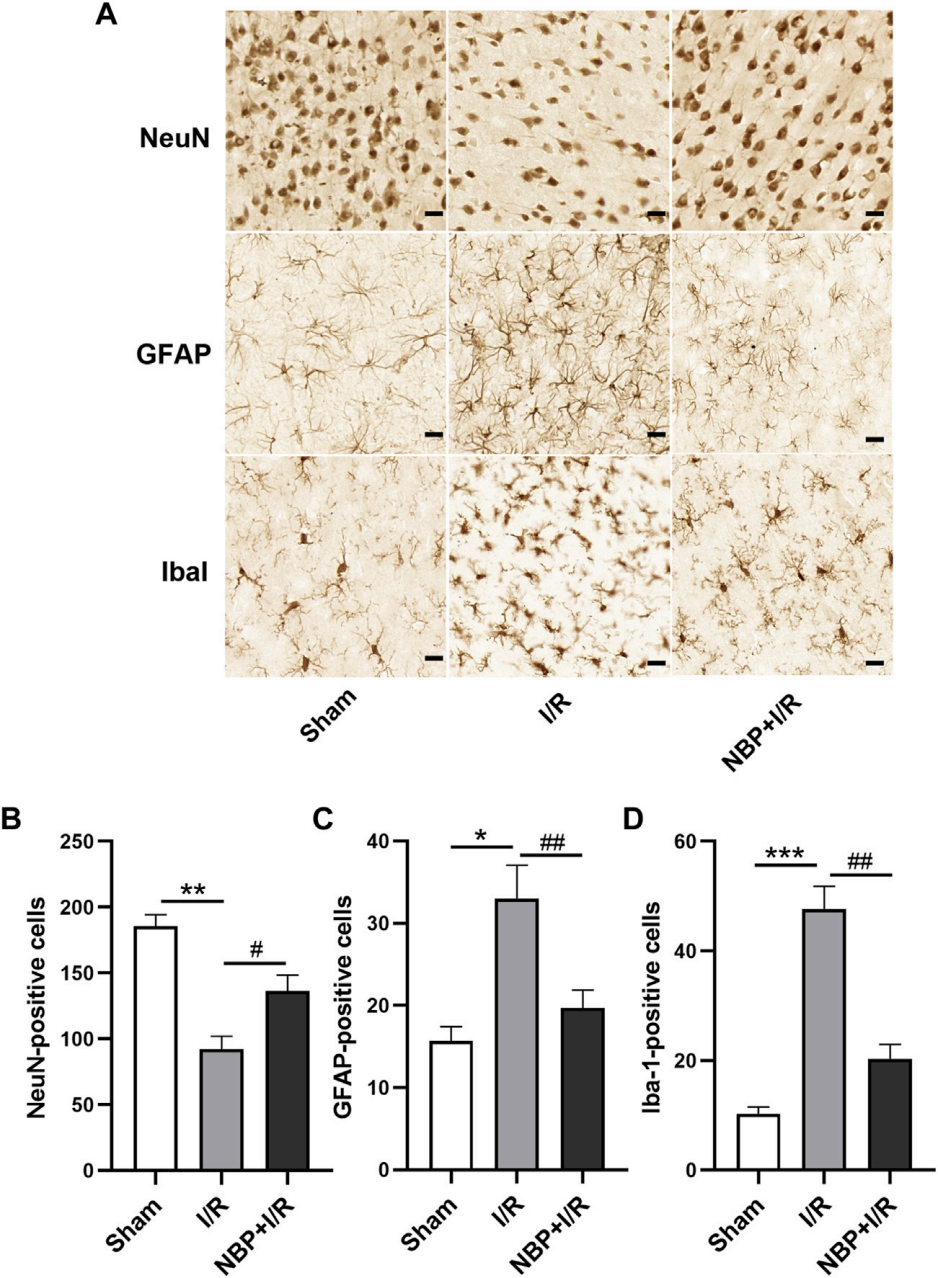
Effects of NBP on nerve cells in the ischemic penumbra in I/R rats. **(A)** NeuN, GFAP and Iba1 staining by immunochemistry (scale bar = 20 μm). NBP significantly increased NeuN-positive cells **(B)** and decreased GFAP-positive cells **(C)** and Iba1-positive cells **(D)**, suggesting that NBP could preserve the viability of neurons and protect microglia and astrocytes against overactivation in I/R rats. Data are expressed as the mean ± SEM (n = 3) and were analyzed by one-way ANOVA (*post hoc* analysis: Tukey’s *post hoc* analysis test). ** (*p* < .01) represents significance compared to the sham group. # (*p* < .05) represents significance compared to the I/R group (*p* < .01). NBP dose: NBP + I/R, 9 mg/kg.

According to the immunohistochemistry results, neuron-specific nuclear protein (NeuN)-positive cells were significantly decreased in the I/R group compared with the sham group (*p* < .01). NBP significantly increased the number of NeuN-positive cells in the ischemic penumbra zone (*p* < .05) (Figures 6A, B), suggesting that NBP could preserve the viability of neurons in I/R rats. Glial fibrillary acidic protein (GFAP) and ionized calcium binding adaptor molecule 1 (Iba1) were analyzed to verify the activation of microglia and astrocytes in the ischemic penumbra, respectively. Compared with the sham group, GFAP-positive cells and Iba1-positive cells were significantly increased in the I/R group (*p* < .01). NBP significantly decreased GFAP-positive cells and Iba1-positive cells (*p* < .05), indicating that NBP could effectively protect microglia and astrocytes against overactivation (Figures 6A, C, D).

## Discussion

At the beginning of ischemic stroke, an irreversible necrotic zone in the brain is first formed, followed by second-phase damage in the peri-infarct area surrounding the core (Darwish et al., 2020). The peri-infarct area, which is called the ischemic penumbra, plays an important role in the clinical deficits of ischemic stroke and can be recovered by rapid reperfusion or neuroprotective treatment (Belayev et al., 2018). PSCI developing after stroke usually leads to worse clinical outcomes (Douven et al., 2018). Compared with healthy controls, ischemic stroke patients with even mild neurological impairment exhibit PSCI, including depressive symptoms, fading memory and decreased social cognitive performance (Pedroso et al., 2018). The pathogenesis of ischemic stroke is quite complex. It has been suggested that the injured brain following ischemic stroke is a functional unit to be protected, and neuroprotective reagents with multiple targets might be a promising option for PSCI treatment (Terasaki et al., 2014).

Our study showed that NBP profoundly ameliorated depression-like disorder, enhanced study and memory competence and promotes social cognition as judged by the SPT, NOR test and SI test, respectively. The protective effect of NBP on neurological function was evaluated in our study. According to neurological scoring and TTC staining results, NBP potently attenuated neurological deficits and reduced infarct volume, consistent with previously reported studies (Zhang et al., 2012; Li et al., 2019). In addition, by laser speckle imaging, we confirmed that NBP was able to enhance CBF in the ischemic cerebral hemisphere in I/R rats, which has been reported to be responsible for neurological deficits (El Amki and Wegener, 2017). Loss of neurons, and excessive activation of microglia and astrocytes play vital roles in cerebral injury and cognitive dysfunction after ischemic stroke (Chamorro et al., 2016; Xu et al., 2017). Suppression of microglia and astrocyte activation and protection of neuronal viability contribute to functional recovery in brain (Wang et al., 2022). In our study, we showed that NBP significantly suppressed the activation of microglia and astrocytes and protected neurons in the ischemic penumbra in I/R rats, indicating that NBP is an effective neuroprotective reagent.

It has been demonstrated that NBP is a multitarget neuroprotectant that suppresses neuroinflammation, reduces oxidative stress, improves mitochondrial function and inhibits neuronal apoptosis (Yang et al., 2015). It is proposed in our study that NBP alleviates PSCI by targeting multiple signaling pathways. Oxidative stress plays a pivotal role in the progression of ischemic stroke by initiating a series of biochemical cascades. ROS are essential signaling molecules that are overexpressed during cerebral ischemia and reperfusion and cause cellular damage and death (Li W et al., 2021). MDA, a kind of lipid peroxide, is another major oxidative stress indicator reflecting the degree of lipid peroxidation and cell damage in the injured brain (Hua et al., 2015). In our study, we show that NBP significantly inhibits ROS and MDA production, indicating that NBP is a potent antioxidant. Nrf2 is a key regulator in antioxidative response. Nrf2 initiates the transcription of a number of antioxidative genes by binding nucleus antioxidant response elements (AREs) after redox stimulation (Hassanein et al., 2020). The cytoplasmic protein Keap1 is a negative regulator of Nrf2. Overexpression of Keap1 inhibits Nrf2 levels and aggravates oxidative stress induced brain injury. In contrast, inhibition of Keap1 releases Nrf2 to the nucleus, thus activating the antioxidant defense system (Byun and Lee, 2015). Recently, a clinical study showed that NBP exerts a neuroprotective effect on ischemic stroke patients by regulating the Keap1/Nrf2 pathway (Zhang et al., 2022). In addition, activation of the Nrf2 pathway suppresses oxidative stress and further attenuates brain injury and improves PSCI in I/R rats (Zhang et al., 2021). Combined with the above research and our results, it is concluded that the Keap1/Nrf2 pathway is also associated with PSCI alleviation by NBP.

Conversely, increasing evidences has shown that inflammation after ischemic stroke poses a second wave of damage to the brain (Veltkamp and Gill, 2016). Neuroinflammation provides potential targets for the treatment of neurological diseases (Chopra et al., 2010; Sharma et al., 2019). It has been proved in both clinical studies and animal experiments that the NF-κB signaling pathway plays an important role in inflammation-induced injury in ischemic stroke (Ran et al., 2021). The involvement of the NF-κB pathway in ischemic stroke makes it an attractive target for the development of anti-inflammatory drugs to treat ischemia-reperfusion injury (Howell and Bidwell, 2020). Our data showed that the levels of inflammatory cytokines, including IL-6, IL-1β and TNF-α, were significantly increased and that NF-κB and iNOS were significantly overexpressed in I/R rats. NBP significantly downregulated the cytokine, NF-κB protein and iNOS protein expression levels, indicating that the NF-κB/iNOS pathway is another important target for NBP.

## Conclusion

In summary, our data suggest that NBP effectively protects neurological function and alleviates PSCI in I/R rats by synergistically suppressing inflammation and oxidative stress by targeting the Keap1/Nrf2 pathway and NF-κB/iNOS pathway, respectively. Therefore, NBP is a promising neuroprotective agent for PSCI treatment.

## Data Availability

The original contributions presented in the study are included in the article/supplementary material, further inquiries can be directed to the corresponding authors.
